# MFAP4-Mediated Effects in Elastic Fiber Homeostasis, Integrin Signaling and Cancer, and Its Role in Teleost Fish

**DOI:** 10.3390/cells11132115

**Published:** 2022-07-05

**Authors:** Ali Mohammadi, Grith L. Sorensen, Bartosz Pilecki

**Affiliations:** Department of Cancer and Inflammation Research, Institute of Molecular Medicine, University of Southern Denmark, 5000 Odense, Denmark; amohammadi@health.sdu.dk (A.M.); glsorensen@health.sdu.dk (G.L.S.)

**Keywords:** cancer, elastic fiber, extracellular matrix, fibrosis, integrin signaling, MFAP4

## Abstract

Microfibrillar-associated protein 4 (MFAP4) is an extracellular matrix (ECM) protein belonging to the fibrinogen-related domain superfamily. MFAP4 is highly expressed in elastin-rich tissues such as lung, blood vessels and skin. MFAP4 is involved in organization of the ECM, regulating proper elastic fiber assembly. On the other hand, during pathology MFAP4 actively contributes to disease development and progression due to its interactions with RGD-dependent integrin receptors. Both tissue expression and circulating MFAP4 levels are associated with various disorders, including liver fibrosis and cancer. In other experimental models, such as teleost fish, MFAP4 appears to participate in host defense as a macrophage-specific innate immune molecule. The aim of this review is to summarize the accumulating evidence that indicates the importance of MFAP4 in homeostasis as well as pathological conditions, discuss its known biological functions with special focus on elastic fiber assembly, integrin signaling and cancer, as well as describe the reported functions of non-mammalian MFAP4 in fish. Overall, our work provides a comprehensive overview on the role of MFAP4 in health and disease.

## 1. Elastic Fiber Structure and Assembly

Extracellular matrix (ECM) is a three-dimensional macromolecular network that supports the surrounding cells [1]. While the exact composition of the ECM varies greatly across different tissues [2], it is predominantly composed of glycoproteins and fibrous macromolecules such as collagens, elastin and proteoglycans. The main function of the ECM is to provide structural and mechanical support to the surrounding tissue. Furthermore, ECM communication with the neighboring cells is essential for morphogenesis, cell adhesion, differentiation and tissue homeostasis [3,4]. The ECM regulates the cellular phenotype and functions either through modulating the bioavailability of extracellular mediators, such as cytokines, growth factors and enzymes, or through directly interacting with cell surface receptors and initiating intracellular signal transduction pathways leading to changes in gene transcription [5,6].

Elastic fibers are large, insoluble ECM structures that provide resilience and elasticity to tissues that undergo repeated stretch, such as blood vessels, lung and skin [7]. Two main structural components of elastic fibers are elastin and fibrillins [5]. The amorphous elastin core comprises the center of the fiber and is surrounded by the fibrillin-rich microfibril sheath [8]. Elastin is a highly hydrophobic polymer of the soluble precursor protein tropoelastin [9]. Under physiological conditions, extracellular tropoelastin undergoes a self-assembly process called coacervation, driven by hydrophobic domain interactions [10]. Coacervation concentrates and properly aligns tropoelastin molecules and constitutes a first step in elastic fiber maturation [11]. Due to the high content of lysine residues, further tropoelastin assembly into a polymeric form is stabilized by formation of desmosine cross-links, rendering mature elastin insoluble [12]. Elastin cross-linking is catalyzed by the lysyl oxidase (LOX) protein family [13].

The other major components of elastic fibers are microfibrils, which provide the structural scaffold for a deposition of elastin globules. Microfibrils consist primarily of fibrillin-1 and fibrillin-2, large tropoelastin-binding glycoproteins [14]. Apart from their structural role as elastin-binding proteins, fibrillins also possess an ability to sequester and regulate the bioavailability of transforming growth factor-β (TGF-β) family members [15]. Moreover, several other ECM proteins are required for proper elastic fiber assembly and function [16,17].

In a multi-step process of elastogenesis, tightly regulated both spatially and temporally, fibrillins assemble into a macromolecular microfibril scaffold onto which elastin is deposited. Assembly of fibrillin monomers into microfibrils takes place at the cell surface and requires involvement of cell surface receptors, such as integrins and heparan sulfate proteoglycans as well as fibronectin [18,19]. After coacervation, elastin microaggregates are stabilized by LOX-mediated cross-linking and transferred onto pre-existing microfibrils [20]. Deposited elastin globules then coalesce and are further cross-linked to form a complete, fully functional fiber [21].

Among the accessory proteins responsible for elastic fiber formation and maintenance, five microfibrillar-associated proteins (MFAPs) have been identified [17]. MFAP2 (also called MAGP-1) and MFAP5 (or MAGP-2) are related proteins involved in elastic fiber assembly [22,23,24]. Gene inactivation studies revealed that MFAP2 regulates processes such as skeletal development, hemostasis and lipid uptake as consequence of its control of TGF-β bioavailability [25,26,27]. MFAP5 can interact with multiple proteins of the Notch signaling pathway and plays an essential role in regulating cell adhesion, angiogenesis and vessel development [28,29,30]. While the name suggests its extracellular location and association with microfibrils, MFAP1 is an intrinsically disordered nucleolar protein orthologous to the yeast SPP381 splicing factor that participates in pre-mRNA processing [31] and has been suggested to regulate cell proliferation and genome integrity [32,33]. *MFAP3* was identified as a candidate gene for heritable microfibril diseases [34], but its role has not been validated so far. Another protein named MFAP3-like (MFAP3L) is a nuclear protein kinase implicated in colorectal cancer invasion and metastasis [35,36,37]. Finally, MFAP4 shares location with other extracellular microfibril-associated proteins but is not structurally related to other MFAPs and belongs to the fibrinogen-related domain (FReD) family [38]. In recent years, MFAP4 has been consistently suggested as a novel regulator of proper tissue homeostasis as well as an important contributor to various pathological processes. In this review, we describe the structure and expression pattern of MFAP4 and summarize its functions in physiological as well as pathological conditions. We focus particularly on the role of MFAP4 in elastic fiber formation, integrin signaling and cancer. Thus, we provide a comprehensive overview of the current knowledge on the biological role of MFAP4.

## 2. FReD Protein Superfamily

FReD-containing proteins are named after vertebrate fibrinogen, the precursor molecule of fibrin in which the domain was first identified [39]. FReDs are independently folding, globular C-terminal domains of 220–250 amino acid residues characterized by more than 40 highly conserved, mostly hydrophobic residues [40]. The FReDs are composed of three distinguishable subdomains: A, B and P. The subdomain A comprises around 50 residues and possesses a single disulfide bond. The B and P subdomains are tightly associated and together consist of 150–200 residues. The C-terminal P subdomain contains a unique disulfide bond and a recognizable ligand binding site. While the FReD of fibrinogen is involved in fibrin clot formation, the predominant function of most other FReD-containing proteins is interaction with cell surfaces of either host or foreign origin [41].

In the human genome, 23 different FReD genes have been identified [38], including three that give rise to fibrinogen, four tenascins, three ficolins, three angiopoietins, six angiopoietin-like proteins and four others: fibrinogen-like protein 1 (hepassocin), fibrinogen-like protein 2 (fibroleukin), fibrinogen domain C-containing protein 1 (FIBCD1) and MFAP4. Among these, MFAP4 shares the highest homology with FIBCD1 and the ficolins. FIBCD1 is an epithelial-restricted transmembrane receptor predominantly expressed in the gut that regulates intestinal inflammation and anti-microbial responses [42]. Ficolins are soluble proteins responsible for host defense against pathogens through multiple mechanisms, including activation of the complement pathway and stimulation of the pro-inflammatory and anti-pathogenic properties of immune cells [43]. Other FReD proteins play a role in various processes including coagulation, development, angiogenesis, wound repair and cell activation [44,45,46].

## 3. MFAP4 Identification and Structure

**Figure 1:**
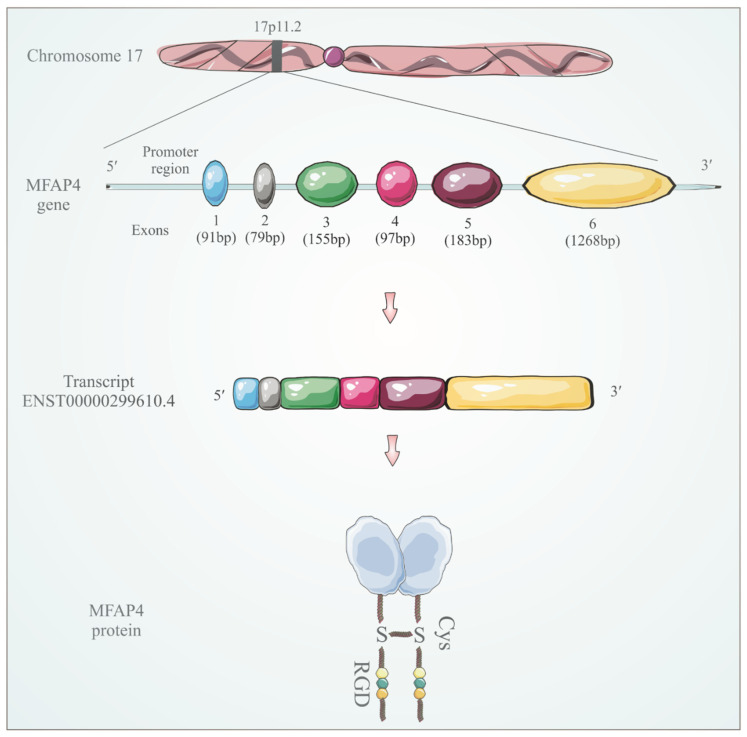
Representation of the *MFAP4* gene structure based on *MFAP4*-201 transcript (ENST00000299610.4). The *MFAP4* gene is located on chromosome 17 and comprises six exons. It is translated to MFAP4 dimeric protein containing N-terminal RGD motif and C-terminal globular fibrinogen-related domain (blue).

MFAP4, also called 36-kDa microfibril-associated glycoprotein (MAGP-36) in some species, was first identified in 1989 in porcine aorta [47]. *MFAP4* gene is located on chromosome 17p11.2 (Figure 1) and was first described in humans as one of the genes commonly deleted in Smith-Magenis syndrome (SMS), a complex genetic disorder characterized by physical abnormalities and neurobehavioral features [48]. SMS is caused by a hemizygous deletion of approximately 5 Mb of chromosome 17p11.2. Although the deletion covers several genes, it has been suggested that the loss of retinoic acid-induced 1 protein is responsible for most of the SMS phenotype [49], whereas MFAP4 does not seem to significantly influence SMS pathology.

The *MFAP4* gene is preceded by a TATA-less promoter that appears to be regulated by coenzyme Q10 and retinol [50]. *MFAP4* gene encodes the 36-kDa protein consisting of a signal peptide, a short N-terminal region containing an Arg-Gly-Asp (RGD) sequence that is a recognition motif for a specific subset of integrin receptors and a C-terminal FReD (Figure 1) [48]. MFAP4 exists as a 66-kDa disulfide-linked dimer that further oligomerizes into octamers [51].

## 4. MFAP4 Localization and Tissue Expression

MFAP4 is abundantly expressed in elastin-rich tissues such as skin, arteries, lung and heart and localizes predominantly within the elastic fibers as seen by immunohistochemistry [52,53]. Using immunogold-labeled electron microscopy, it was demonstrated that MFAP4 localizes to the interface between the microfibrils and the elastin core of elastic fibers but not microfibrils away from the elastin core [54,55]. In the skin, MFAP4 colocalized with elastic fibers in the dermis but not the epidermis, and MFAP4 synthesis was demonstrated in dermal fibroblasts in vivo and keratinocytes in vitro [56,57]. In the lung, MFAP4 immunoreactivity was shown in the interalveolar septae and pulmonary arterioles. MFAP4 was also detected as a soluble protein in bronchoalveolar lavage fluid, where it was shown to interact with surfactant proteins A and D [51,58]. In the heart, non-myocyte cells are the primary source of MFAP4 protein expression that is further increased upon TGF-β1 activation [59]. Additionally, MFAP4 is strongly expressed together with other ECM components, such as collagen type I, in a specific fibroblast subpopulation upregulated in heart failure [60].

Schlosser et al. [61] showed that MFAP4 is expressed by contractile vascular smooth muscle cells (SMCs) in the arteries. Mölleken et al. [62] showed that MFAP4 is highly upregulated in cirrhotic liver tissue and suggested the source of synthesis to be hepatic stellate cell-derived myofibroblasts. MFAP4 expression was subsequently shown to colocalize with α-smooth muscle actin, a marker for myofibroblasts, in the fibrotic liver [63]. Overall, MFAP4 expression appears restricted to cells of mesenchymal origin, particularly fibroblasts and SMCs, and the fibroblasts are reported to be a main cellular source of MFAP4 in most tissues [64,65].

## 5. Role of MFAP4 in Elastic Fiber Assembly

**Figure 2:**
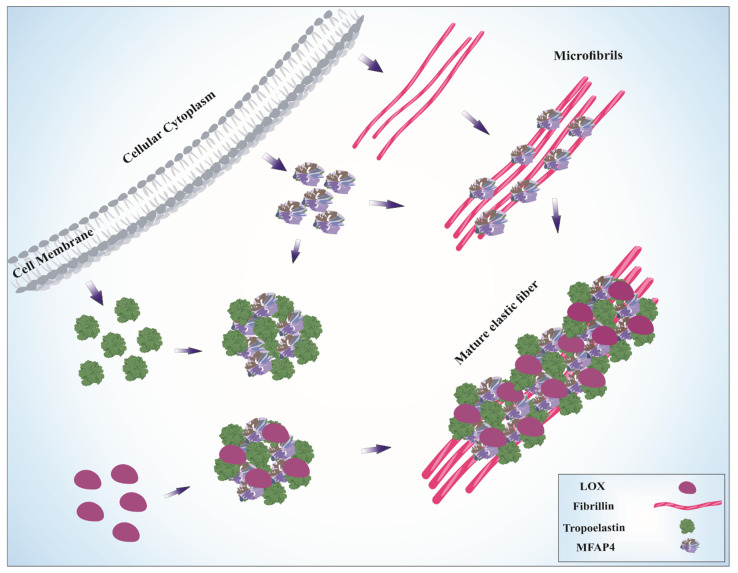
Contribution of MFAP4 to elastic fiber assembly. MFAP4, tropoelastin and fibrillins are secreted to the extracellular space. MFAP4 directly binds to fibrillins and participates in microfibril assembly. MFAP4 also promotes coacervation of single tropoelastin molecules. Tropoelastin then binds to the lysyl oxidase (LOX) and is cross-linked and deposited onto the microfibril scaffold to create a mature elastic fiber. MFAP4 also binds LOX independently from tropoelastin. For clarity, the MFAP4 octamers are presented in a simplified form.

Localization at the microfibril-elastin interface suggests a functional role for MFAP4 in elastogenesis. Indeed, both endogenous and exogenous MFAP4 promotes association between tropoelastin and fibrillin-1 in human dermal fibroblasts. MFAP4 was also shown to accelerate microfibril assembly through direct interactions with fibrillin-1 [57]. MFAP4 interaction with fibrillin-1 was later confirmed in murine skin in vivo by colocalization [66]. MFAP4 was also shown to bind insoluble elastin as well as lysyl oxidase and tropoelastin and facilitate coacervation, the tropoelastin self-assembly process essential for elastic fiber maturation. The interaction between MFAP4 and tropoelastin was calcium-dependent, suggesting the involvement of the FReD domain [66]. Moreover, MFAP4 was demonstrated to disappear in photoaged dermis while accumulating in disintegrated elastic fibers in the lesional skin of pseudoxanthoma elasticum, an elastin-related disorder [56]. In line with that, MFAP4 expression was significantly decreased both in extrinsically photoaged and intrinsically aged human skin in a human skin xenograft mouse model. Furthermore, it was demonstrated that MFAP4 suppresses MMP-1 and MMP-12 activity in vitro and in vivo, protecting against elastin and collagen fiber degradation [57]. These observations support an important role for MFAP4 in elastic fiber formation and stability in the skin (Figure 2). Such relation is further supported by a recent GWAS study showing association between *MFAP4* single nucleotide polymorphism rs139356332-G and youthful appearance [67].

While global *MFAP4* deficiency does not seem to infer strong phenotypic changes [61,68], it was reported that adult *MFAP4*-deficient mice exhibit disturbances in pulmonary architecture, characterized by increased inspiratory capacity, decreased alveolar surface area, lowered parenchymal density and emphysema-like airspace enlargement that progresses with age. The loss of elastic alveolar tissue in *MFAP4*-deficient lungs supports the role of MFAP4 in elastogenesis; however, the relative elastin to total protein content was unaffected by *MFAP4* genotype. On the other hand, airway and pulmonary MFAP4 levels were increased in 8-month-old mice relative to 3-month-old-mice, suggesting a compensatory upregulation aimed at maintaining lung function despite the age-dependent loss of alveolar tissue [68]. Strong upregulation of *MFAP4* expression has also been detected in the lungs of patients with chronic obstructive pulmonary disease, where it correlated to expression of other elastogenesis-related genes including elastin and fibulin-5 [69]. However, *MFAP4* expression was inversely correlated to lung function parameters, suggesting that its roles in homeostasis and during pathogenesis might be distinct.

Further evidence about the role of MFAP4 in elastic tissues comes from studies on Marfan syndrome (MFS), a rare genetic disorder of the connective tissue caused by mutations in the fibrillin-1 gene. One of the common manifestations of MFS is thoracic aortic aneurysm, and both MFAP4 mRNA and protein expression are upregulated in aortic aneurysm tissue from MFS patients. Furthermore, MFAP4 showed increased and more diverse glycosylation in MFS patients compared to healthy controls [70]. These changes in MFAP4 expression and glycosylation patterns were suggested to be a compensatory response to compromised elastic structure of the aortic wall, further underlining MFAP4 contribution to maintenance of proper elastic fiber functions. Taken together, the collected literature strongly suggests that a homeostatic function of MFAP4 is maintenance of elastic tissue integrity.

## 6. MFAP4-Mediated Integrin Signaling

**Figure 3:**
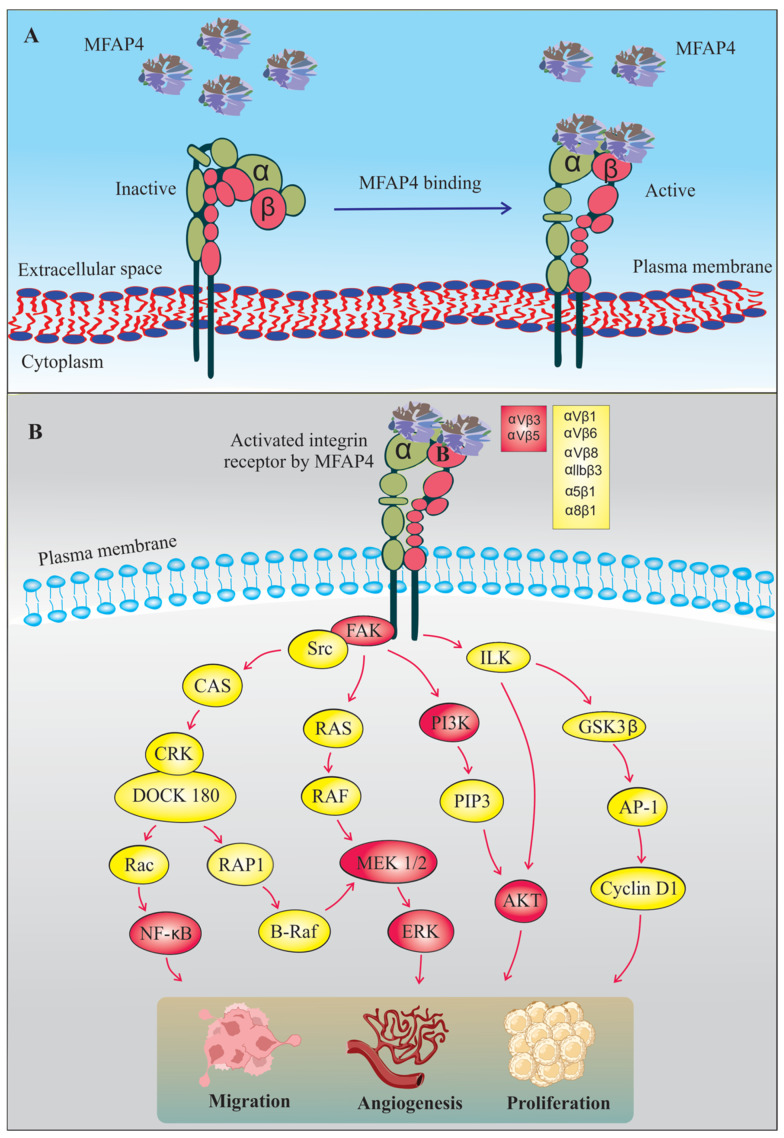
Integrin-dependent signaling secondary to MFAP4 recognition. (**A**) Suggested conformational changes of integrin heterodimer after MFAP4 binding. (**B**) MFAP4-dependent integrin ligation initiates downstream intracellular signaling pathways leading to cellular activation [73,74,75]. Integrin heterodimer pairs and intracellular mediators reported to be MFAP4-dependent are shown in red boxes. Putative integrin heterodimer pairs and intracellular mediators suggested to interact with MFAP4 are shown in yellow boxes. For clarity, the MFAP4 octamers are presented in a simplified form.

Whereas homeostatic effects of MFAP4 appear to be mediated by the FReD domain binding to the ECM fibers, MFAP4 possesses the capacity to modulate pathological processes through independent cellular integrin activation. Integrins are heterodimeric transmembrane receptors composed of α and β subunits. In mammals, twenty-four different integrin subtypes have been identified. Specific integrin dimer subclasses can bind distinct extracellular ligands with varying affinities, and regulate multiple processes including cell adhesion, proliferation, migration, angiogenesis, cytokine production, malignancy, apoptosis, inflammation and tissue repair [71,72]. Integrin signaling is bidirectional, comprising both the inside-out and the outside-in signaling. Outside-in signaling occurs when integrins recognize extracellular ligands leading to activation of focal adhesion kinase (FAK) and integrin-linked kinase (ILK), and consequently to initiation of downstream intracellular signaling cascades [73,74,75] (Figure 3). Inside-out signaling transmits the intracellular signals from the cytoskeleton through the cytoplasmic part of the integrin and regulates its ligand-binding affinity [76].

Due to the presence of a N-terminal RGD motif, MFAP4 can bind to members of a subgroup of RGD-dependent integrins and modulate cellular behavior [55]. While MFAP4-related signaling was shown to increase fibroblast migration, and thus, potentially contribute to physiological processes such as cutaneous wound healing [77], most studies have reported that MFAP4 plays an aggravating role in various pathological contexts. In vascular proliferative disorders, activation of vascular smooth muscle cells constitutes a crucial process regulating vessel wall remodeling. MFAP4 was found to promote vascular SMC proliferation and migration through activation of FAK and downstream mediators including PI3K and ERK after carotid artery ligation-mediated injury as well as in human aortic vascular SMCs in vitro [61]. MFAP4 also mediated integrin-dependent direct monocyte recruitment important in pathological remodeling in both neointima formation and aortic aneurysms [78]. Furthermore, the interplay between MFAP4 and integrin signaling has been associated with cardiac remodeling. *MFAP4* deficiency was shown to attenuate ventricular arrhythmias and cardiac fibrosis induced by pressure overload following aortic banding or isoproterenol administration, caused by inhibition of FAK signaling [79]. Dorn et al. [59] further highlighted the importance of MFAP4-mediated integrin activation by showing regulation of downstream intracellular signaling cascades in cardiac cells in stress-induced cardiac remodeling. However, contrary to previous findings, they reported that *MFAP4*-deficient mice exhibited worsened cardiac function with increased cardiac hypertrophy in short-term models of pressure overload.

MFAP4 interaction with integrins was also shown to be important for bronchial SMC activation [80]. Whereas the main receptor for MFAP4 in the vascular system appears to be integrin αvβ3 [61], integrin αvβ5 is seemingly important in mediating MFAP4-dependent effects in the airways [80]. *MFAP4*-deficient mice were partially protected from all major hallmarks of experimental asthma development—airway hyperresponsiveness, inflammation and pulmonary remodeling. Furthermore, MFAP4 was upregulated in human bronchial SMCs isolated from asthmatics and promoted both asthmatic bronchial SMC proliferation and their expression of eosinophil chemoattractant CCL11 in an integrin αvβ5-dependent manner [80]. Strengthening these observations, circulating MFAP4 was also shown to be mildly upregulated in asthmatic adolescents and young adults [81]. Overall, the deleterious functions of MFAP4 in pathology seem to be uniformly mediated through interaction with RGD-dependent integrins and activation of related downstream signaling. These effects are expected to be mediated by ECM-bound MFAP4, as MFAP4-dependent effects in vitro are evident after cell stimulation with recombinant MFAP4 immobilized at the cell culture surface [61].

## 7. The Role of MFAP4 in Tissue Fibrosis

While circulatory MFAP4 is recognized to vary moderately with cardiovascular disease and mortality and slightly with asthma [81,82,83], it is markedly increased in liver fibrosis and cirrhosis. MFAP4 was identified amongst the highest regulated proteins in cirrhotic septae by proteomic analysis [62]. Accordingly, *MFAP4* gene expression in the liver was upregulated in patients with non-alcoholic fatty liver disease- as well as alcoholic steatohepatitis-related fibrosis [63,84]. Circulatory MFAP4 is likewise increased in patients with hepatic fibrosis caused by both hepatitis C virus infection and alcoholic liver disease and correlates significantly with disease severity [62,84,85]. The increase in circulatory MFAP4 in liver fibrosis was further shown to be significantly associated with transient elastography and chronic viral infection [86]. On this basis, MFAP4 has been proposed as a candidate diagnostic biomarker for disease staging in hepatic fibrosis and cirrhosis.

In the heart, *MFAP4* was upregulated in a model of angiotensin II (Ang II)-induced atrial fibrillation, while *MFAP4*-deficient mice showed reduced atrial enlargement and fibrosis. Moreover, *MFAP4* deficiency inhibited Ang II-induced activation of FAK, PI3K, AKT and ERK kinases [87], indicating that the observed effects were integrin-mediated. In another study, it was reported that in freshly isolated rat cardiac cells, *MFAP4* expression was 40 times higher in cardiac fibroblasts than in cardiomyocytes. Furthermore, stimulation of cardiac fibroblasts with TGF-β or Ang II increased expression of fibrotic mediators including Col3, α-SMA and MFAP4. In line with that, shRNA-based *MFAP4* silencing in TGF-β1-stimulated cardiac fibroblasts resulted in significant downregulation of Col1a1, fibronectin, α-SMA and Col3, and the importance of MFAP4 in activating PI3K, AKT and MEK1/2-ERK1/2 signaling pathways was further validated [79]. In agreement with that, *MFAP4* deficiency attenuated aortic collagen deposition as well as FAK and TGF-β pathway activation in an Ang II-induced abdominal aortic aneurysm model resulting in reduced aneurysm size [78].

MFAP4 has also been linked to fibrotic remodeling in other organs. In the lung, MFAP4 levels were upregulated in the bronchoalveolar lavage fluid as well as the pulmonary ECM in bleomycin-induced pulmonary fibrosis [88,89]. Moreover, *MFAP4* deficiency limited peribronchial fibrosis and reduced total lung collagen content induced by experimental asthma [80]. MFAP4 was also shown to promote renal fibrosis in the unilateral ureteral obstruction mouse model, where it was reported that the renal TGF-β pathway, plasminogen activator inhibitor-1, collagen I and fibronectin were all downregulated in *MFAP4*-deficient mice. Moreover, *MFAP4*-deficient mice showed suppressed renal inflammation caused by inhibition of the NF-κB signaling pathway. These results were confirmed in vitro in the HK-2 kidney epithelial cell line, where *MFAP4* knockdown inhibited NF-κB signaling and downstream expression of pro-inflammatory markers [90]. Taken together, these findings indicate that MFAP4 is an essential activator of integrin-mediated tissue remodeling and fibrosis.

## 8. MFAP4 in Cancer

Significant variation in *MFAP4* expression has been reported in cancer, and MFAP4 has been suggested as a potential prognostic and predictive biomarker in different types of human cancers [91]. However, the exact pattern of MFAP4 regulation and its possible role in distinct cancer types appear contradictive (Table 1).

In prostate and urinary bladder cancers, *MFAP4* level is decreased, and it was consequently suggested to exert tumor-suppressive effects [92,93]. Likewise, Muraoka et al. [94] established that low-risk breast cancer patients have increased MFAP4 protein expression and suggested that MFAP4 can be a novel prognostic marker in the detection of breast cancer. In lung adenocarcinoma, *MFAP4* expression is highly downregulated; moreover, micro-RNA147b was identified to inhibit *MFAP4* expression while promoting tumor cell proliferation, migration and colony formation [95]. Accordingly, decreased *MFAP4* expression in lung adenocarcinoma was confirmed in a separate study, where *MFAP4* overexpression attenuated cell invasion and stemness in vitro and inhibited tumor growth in vivo [96]. Furthermore, high MFAP4 tumor levels have been associated with increased survival [97]. In oral squamous cell carcinoma, *MFAP4* expression was significantly decreased, while high *MFAP4* was associated with better prognosis [98]. Similar results were reported in head and neck squamous cell cancer [99]. On the other hand, in adrenocortical carcinoma, *MFAP4* levels were decreased but did not affect overall survival [100]. These studies all suggest that MFAP4 might work as a tumor suppressor.

In contrast, Yang et al. [91] reported that in advanced stages of breast cancer, stomach cancer and lung cancer, high *MFAP4* levels are associated to poor prognosis. Pathway enrichment analysis suggested that MFAP4 might modulate nucleotide excision repair and DNA damage recognition. Increased serum MFAP4 was observed in hepatocellular carcinoma patients [101,102] and *MFAP4* was suggested as a potential biomarker in the serous ovarian cancer due to high expression and association with chemoresistance [103]. *MFAP4* is also highly expressed in human neuroblastoma, where it was associated with lower survival rates. Furthermore, *MFAP4* was identified as a direct target of a tumor-suppressive micro-RNA449a that participates in neuroblastoma cell differentiation [104]. Increased MFAP4 expression was observed in the pancreatic ductal adenocarcinoma (PDA) stroma, with MFAP4 identified as a carrier of the tumor-associated carbohydrate sialyl-Lewis x, suggesting this MFAP4 glycoform as a potential PDA biomarker [105]. In ovarian cancer patients, low methylation of *MFAP4* gene was correlated with poor progression-free survival [106]. Increased *MFAP4* expression was also reported in leiomyosarcomas [107] as well as pleomorphic adenoma, a benign tumor of salivary glands [108].

The apparently complex role of MFAP4 in cancers is further supported by the Human Protein Atlas database [65] showing clinical survival analyses in which local tumor MFAP4 expression can be either favorable or unfavorable depending on the cancer type (unfavorable: cervical, endometrial, glioma, melanoma, ovarian, renal, stomach, thyroid and urothelial cancer; favorable: breast, head and neck, liver, lung, pancreatic and prostate cancer) [109].

All in all, existing literature reports opposing changes in MFAP4 expression in different cancer types, possibly reflecting the tissue-specific characteristics. Interestingly, MFAP4 upregulation in cancer is often reported in tissues of low stiffness, such as neural tissue and blood. It has been suggested that the role of MFAP4 in cancer might be dual, with MFAP4 acting as a tumor suppressor facilitating inflammatory cell recruitment and immunological surveillance in early-stage cancer, while promoting cell proliferation and migration in later, advanced stages. MFAP4 might also serve different functions depending on its location within the cancer and the surrounding stroma. Therefore, more mechanistic studies are needed to determine the exact role of MFAP4 in cancer pathogenesis, considering the cellular and histological differences between the distinct disease states.

**Table 1:** Regulation of MFAP4 expression in different cancer types.

References	Sample Size	Type of Analysis	MFAP4 Expression	Sample Type	Cancer Type
[91]	114 normal cases 1097 primary tumors	Bioinformatic (TCGA)	Downregulation	mRNA	Breast cancer
[94]	27 patients	Tissue samples	Downregulation	Protein	Breast cancer
[91]	7 different datasets	Bioinformatic (TCGA)	Downregulation	mRNA	Bladder cancer
[91]	8 different datasets	Bioinformatic (TCGA)	Downregulation	mRNA	Colorectal cancer
[91]	1 dataset	Bioinformatic (TCGA)	Downregulation	mRNA	Cervical cancer
[91]	5 different datasets	Bioinformatic (TCGA)	Downregulation	mRNA	Head and neck cancer
[91]	6 different datasets	Bioinformatic (TCGA)	Downregulation	mRNA	Kidney cancer
[91]	574 cases	UALCAN database	Downregulation	mRNA	Lung cancer
[95,96]	59 normal cases 535 tumor cases	Bioinformatic (TCGA)	Downregulation	mRNA	Lung adenocarcinoma
[91]	421 cases	UALCAN database	Downregulation	mRNA	Liver cancer
[91]	3 different datasets	Bioinformatic (TCGA)	Downregulation	mRNA	Ovarian cancer
[98]	22 normal cases 57 tumor cases	Bioinformatic (GSE25099)	Downregulation	mRNA	Oral squamous cell carcinoma
[92]	19 prostate cancers 33 benign prostate hyperplasia	Tissue Samples	Downregulation	Protein	Prostate cancer
[91]	449 cases	Bioinformatic (UALCAN)	Downregulation	mRNA	Stomach cancer
[93]	10 urinary bladder cancers 5 normal tissue samples	Tissue Samples	Downregulation	mRNA	Urinary bladder cancer
[91]	2 different datasets	Bioinformatic (TCGA)	Upregulation	mRNA	Brain cancer
[91]	2 different datasets	Bioinformatic (TCGA)	Upregulation	mRNA	Esophageal cancer
[102]	50 patients	Serum samples	Upregulation	Protein	Hepatocellular carcinoma
[91]	2 different datasets	Bioinformatic (TCGA)	Upregulation	mRNA	Leukemia
[91]	4 different datasets	Bioinformatic (TCGA)	Upregulation	mRNA	Lymphoma
[104]	476 patients	Cohort study	Upregulation	mRNA	Neuroblastoma
[105]	25 pancreatic ductal adenocarcinomas 3 control patients	Tissue Samples	Upregulation	Protein	Pancreatic adenocarcinoma
[108]	15 pleomorphic adenomas 15 adjacent normal tissues	Tissue Samples	Upregulation	mRNA	Pleomorphic adenoma

## 9. MFAP4 and Its Role in Teleost Fish

Besides mammals, teleost fish are often used to model human development, homeostasis and disease mechanisms [110,111]. The role of MFAP4 has also been extensively studied in teleost fish. While humans and other mammals possess a single full-length MFAP4 molecule encoded by a single gene, there are multiple *mfap4* genes in teleost fish [112]. Catfish *mfap4* genes show low sequence similarity to human *MFAP4*, ranging from 36 to 51%. Moreover, teleost MFAP4s lack a RGD sequence responsible for integrin recognition, suggesting that their functions might be distinct from these reported for mammalian MFAP4 [113].

In zebrafish embryos, *mfap4* showed an expression pattern typical for early macrophages, and its expression was abolished in embryos with knockdown od *spi1*, a transcription factor crucial for myeloid and lymphoid cell development. The expression of *mfap4* colocalized completely with macrophage marker *csf1* but did not overlap with neutrophil-specific marker *mpx* and was absent in the thymus. Based on that, MFAP4 was identified as a specific and robust macrophage lineage marker [114] and has since been established as a stable and robust marker or promoter driving the macrophage-specific gene expression [115,116,117]. Moreover, it was shown that MFAP4 plays a role in hematopoiesis and development of myeloid cells, with *mfap4*-deficient fish exhibiting lowered macrophage numbers with a concomitant increase in neutrophils and altered expression of lineage-determining transcription factors [118], underlining the importance of MFAP4 for proper macrophage differentiation in teleosts.

In catfish, a strong *mfap4* upregulation was reported after infection with Gram-negative bacterium *Edwardsiella ictalurid* [119]. Accordingly, five distinct *mfap4* mRNAs were subsequently reported to be rapidly and strongly upregulated upon bacterial infection with *Edwardsiella ictaluri* or *Flavobacterium columnare*. While all five *mfap4* transcripts were widely expressed across the catfish tissues, they clustered into two distinct expression patterns. *Mfap-1, -3* and *-5* were expressed predominantly in the gills and skin, while the highest expression of *mfap-2* and *-4* was observed in the liver and muscle [113]. In Nile tilapia, basal *mfap4* expression was the most prominent in the liver and intestine. After bacterial challenge with *Streptococcus agalactiae* or *Aeromonas hydrophila*, *mfap4* expression was rapidly upregulated in the liver, spleen and head kidney, the primary organs attacked by bacterial infections, as well as in vitro in isolated monocytes/macrophages. Recombinant *mfap4* was also shown to bind and agglutinate the bacteria, promote phagocytosis and upregulate the expression of pro-inflammatory cytokines, including IL-1β, IL-6 and TNF [120]. These data point towards the importance of catfish MFAP4 in pattern recognition and innate immune defense against invading pathogens. Interestingly, teleost fish lack ficolins, FReD proteins mediating innate immunity through pathogen recognition and complement activation, and it is possible that piscine MFAP4 molecules mediate similar functions in the absence of ficolin orthologs in fish.

MFAP4 has also been implicated in other processes. It was reported among the differentially expressed genes in the gonads of the female rare minnow after stimulation with synthetic androgen 17α-methyltestosterone [121], implying its potential involvement in ovarian development. In another RNAseq study, *mfap4* was downregulated in the carp-goldfish transparent mutants relative to WT fish, with an accompanying upregulation of *mfap4*-targeting miRNA-146a, suggesting its association to pigmentation [122]. However, these isolated observations remain to be validated in future studies.

In summary, teleost MFAP4 apparently functions as an innate immune molecule expressed by macrophages, which is a different role and expression profile from those described for mammalian MFAP4. Human MFAP4 has the highest homology with FIBCD1 and the ficolins that all are FReD-containing pattern recognition receptors [123]. Such a role is yet unexplored for human MFAP4 and remains to be demonstrated, although a demonstration of mannan-binding lectin activity of soluble MFAP4 and its ability to interact with lung collectins SP-A and SP-D might reflect such pattern recognition functions [58].

## 10. Conclusions

Mammalian MFAP4 is an ECM microfibril-associated protein abundantly present in elastic tissues. During homeostasis, MFAP4 regulates proper elastin fiber organization and function through the FReD domain, thus supporting tissue integrity of elastin-rich tissues, such as skin and lung. However, in the pathological setting MFAP4 can elicit integrin-related signaling, contributing to development and progression of diseases such as cardiovascular disorders, asthma and fibrosis. While MFAP4 expressional levels are often, but not always, regulated during disease, the circulatory MFAP4 levels are significantly correlated with liver fibrosis staging and have potential as a diagnostic biomarker for liver fibrosis and cirrhosis. In cancer, MFAP4 seems to be differentially regulated depending on the specific cancer type and location. Finally, data generated in teleost fish suggest that MFAP4 might contribute to macrophage differentiation and innate immune defense. All in all, MFAP4 is emerging as an important contributor to several essential biological processes.

## 11. Future Perspectives

While MFAP4 research in the recent years has elucidated many of its biological functions, several of the MFAP4-mediated effects have not been explained or studied in detail. MFAP4 expression pattern should be analyzed further, focusing on identifying the specific MFAP4-expressing cell subpopulations, particularly those arising in diseased conditions. The potential of MFAP4 as a disease biomarker in tissues other than the liver remains to be established. It is also of interest to investigate if some of the macrophage-specific immune-regulating teleost MFAP4 functions are conserved in humans. Finally, characterization of the exact role of MFAP4 in the pathogenesis of cancer and other conditions requires future mechanistic studies. Overall, while further investigations are warranted to address these yet unexplored issues of MFAP4 biology, the development of novel MFAP4-targeting strategies inhibiting its interaction with integrins may putatively be effective as therapeutic tools in diseases such as asthma, cardiovascular disorders, fibrosis and cancer.
